# Hydrogen-Deuterium Exchange Profiles of Polyubiquitin Fibrils

**DOI:** 10.3390/polym10030240

**Published:** 2018-02-27

**Authors:** Daichi Morimoto, Ryo Nishizawa, Erik Walinda, Shingo Takashima, Kenji Sugase, Masahiro Shirakawa

**Affiliations:** 1 Department of Molecular Engineering, Graduate School of Engineering, Kyoto University, Kyoto-Daigaku Katsura, Nishikyo-Ku, Kyoto 615-8510, Japan; morimoto@moleng.kyoto-u.ac.jp (D.M.); ryo.nishizawa@sumitomocorp.com (R.N.); takashima.shingo.44x@st.kyoto-u.ac.jp (S.T.); 2 Department of Molecular and Cellular Physiology, Graduate School of Medicine, Kyoto University, Yoshida Konoe-cho, Sakyo-ku, Kyoto 606-8501, Japan; walinda.erik.6e@kyoto-u.ac.jp

**Keywords:** ubiquitin, amyloid fibrils, hydrogen-deuterium exchange

## Abstract

Ubiquitin and its polymeric forms are conjugated to intracellular proteins to regulate diverse intracellular processes. Intriguingly, polyubiquitin has also been identified as a component of pathological protein aggregates associated with Alzheimer’s disease and other neurodegenerative disorders. We recently found that polyubiquitin can form amyloid-like fibrils, and that these fibrillar aggregates can be degraded by macroautophagy. Although the structural properties appear to function in recognition of the fibrils, no structural information on polyubiquitin fibrils has been reported so far. Here, we identify the core of M1-linked diubiquitin fibrils from hydrogen-deuterium exchange experiments using solution nuclear magnetic resonance (NMR) spectroscopy. Intriguingly, intrinsically flexible regions became highly solvent-protected in the fibril structure. These results indicate that polyubiquitin fibrils are formed by inter-molecular interactions between relatively flexible structural components, including the loops and edges of secondary structure elements.

## 1. Introduction

Ubiquitin is a post-translational modifier, and covalent modification of proteins with ubiquitin (ubiquitylation) is related to myriad biological processes, including cell cycle progression and immune response [1]. Physicochemically, ubiquitin is known to be an extremely stable and rigid protein; however, it is often found in intracellular aggregates associated with neurodegenerative disorders such as Alzheimer’s and Parkinson’s diseases [2,3]. We previously found that the thermodynamic stability of ubiquitin decreases as a function of the degree of polymerization, and that polyubiquitin forms amyloid-like fibrils upon application of heat or shear stress [4]. Furthermore, in living cells, polyubiquitin also forms fibrillar aggregates that can be selectively degraded by macroautophagy [4,5]. Thus, in cells, ubiquitylation is related to the formation of fibrillar aggregates that can be substrates for macroautophagy; however, in the case of macroautophagy dysfunction, such aggregates accumulate, which can contribute to the development of neurodegenerative diseases [6]. In the proteolytic clearance of the polyubiquitin fibrillar aggregates, the structure of the fibrils may play a key role, possibly facilitating recognition by receptor proteins of the macroautophagy system; nevertheless, structural details of polyubiquitin fibrils remain scarce.

To investigate the atomic-level structures of amyloid fibrils, solid-state NMR spectroscopy, electron microscopy, and X-ray crystallography have been utilized so far. In addition, the hydrogen-deuterium (HD) exchange technique has been used to acquire structural information on the solvent-protection of the fibrils [7]. In this study, we focused on fibrils composed of M1-linked diubiquitin and identified their solvent-protection profiles by the HD exchange method using solution NMR spectroscopy. Diubiquitin is the shortest possible form of polyubiquitin, and M1-linked diubiquitin forms amyloid-like fibrils most readily [4]. Therefore, this molecule offers a simple approach for the investigation of the structural properties of polyubiquitin fibrils.

## 2. Materials and Methods

### 2.1. Protein Sample Preparation

A C-terminal G75A/G76A mutant of human ubiquitin (Ub^G75A/G76A^) and *N*-terminal glutathione *S*-transferase (GST)-tagged ubiquitin were expressed in *Escherichia coli* strain BL21 (*DE3*) in LB media (Nacalai Tesque, Kyoto, Japan) or M9 minimal media containing 99% ^15^N-labeled ammonium chloride (Cambridge Isotope Laboratories, Tewksbury, MA, USA) with 99% U-^13^C-labeled d-glucose (Cambridge Isotope Laboratories) or unlabeled d-glucose (Nacalai Tesque). Ub^G75A/G76A^ was purified as described previously [8]. GST-tagged ubiquitin was purified by glutathione-affinity chromatography (Glutathione Sepharose 4 Fast Flow, GE Healthcare, Buckinghamshire, UK) in a buffer containing 50 mM Tris-HCl, 200 mM sodium chloride, 1 mM DTT, pH 8.0. The *N*-terminal GST tag was cleaved by HRV3C protease and the tag-free ubiquitin contained the additional *N*-terminal sequence GPLG (^GPLG^Ub). Using unlabeled or ^15^N-labeled Ub^G75A/G76A^ and ^GPLG^Ub, M1-linked diubiquitin was synthesized by enzymatic reaction using E1 (Uba1), E2 (UbcH7), and E3 (HOIL-1L-HOIP complex) enzymes as described previously [4]. The plasmid of the E1 enzyme was a gift from Jorge Eduardo Azevedo (Addgene plasmid #32534). The plasmids of E2 and E3 enzymes were obtained from Kazuhiro Iwai (Kyoto University, Kyoto, Japan). Diubiquitin and monoubiquitin were separated by cation exchange chromatography (Source S, GE Healthcare) as shown previously [9]. Protein purity was checked by sodium dodecyl sulfate polyacrylamide gel electrophoresis (SDS-PAGE).

### 2.2. Hydrogen-Deuterium Exchange Experiments

To obtain M1-linked diubiquitin fibrils, 0.3 mL of a 0.05 mM solution of M1-linked diubiquitin was incubated at 363 K for 15 min in phosphate buffer (50 mM sodium phosphate, 100 mM sodium chloride, pH 6.98) as described previously [4]. The solution was centrifuged at 20,000 *g* for 10 min at 277 K. Next, the fibrils were washed with ice-cold D_2_O (Cambridge Isotope Laboratories)-based phosphate buffer. This washing process was repeated twice and the fibrils were suspended in 0.3 mL of H_2_O or D_2_O-based phosphate buffer. The solution was incubated at 310 K for 24 h, followed by centrifugation at 20,000 *g* for 10 min at 277 K. After twice washing with ice-cold D_2_O, the fibrils were suspended in D_2_O, frozen with liquid nitrogen, and lyophilized. The dried fibrils were dissolved in 0.03 mL of d_6_-dimethyl sulfoxide (DMSO, Sigma Aldrich, St. Louis, MO, USA) containing 0.26% (*v/v*) d_1_-trifluoroacetic acid (TFA, Sigma Aldrich) and immediately diluted to 0.3 mL with d_6_-DMSO to obtain a 0.026% (*v/v*) d_1_-TFA solution. The TFA concentration for complete dissolution of diubiquitin in DMSO was empirically determined.

### 2.3. NMR Spectroscopy

All NMR spectra were acquired on an Avance 600 or 700 MHz NMR spectrometer equipped with a 5 mm ^15^N/^13^C/^1^H *z*-gradient triple resonance cryoprobe (Bruker BioSpin, Rheinstetten, Germany). For hydrogen-deuterium (HD) exchange experiments in the native state, the sample was dissolved in D_2_O-based buffer (50 mM sodium phosphate, 100 mM sodium chloride, pH 6.98). To evaluate HD exchange protection of the M1-linked diubiquitin fibrils, ^1^H-^15^N HSQC spectra of unfolded M1-linked diubiquitin in DMSO were acquired at 298 K. Peak intensities were normalized with the peak volume of methyl protons (0–1.8 ppm) of the same sample as obtained from a water-suppressed ^1^H NMR spectrum using the excitation sculpting pulse scheme [10]. For sequential backbone assignments, ^1^H-^15^N HSQC, HNCACB [11], CBCA(CO)NH [12], and (H)N(COCO)NH [13] spectra were acquired at 298 K. ^1^H chemical shifts were referenced with respect to sodium 2,2-dimethyl-2-silapentane-5-sulfonate (DSS, Tokyo Chemical Industry, Tokyo, Japan) and both ^13^C and ^15^N chemical shifts were calibrated indirectly [14]. ^1^H-^15^N resonance assignments for each subunit of diubiquitin in the native state were derived from entry 17,769 in the Biological Magnetic Resonance Bank. The concentration of monoubiquitin for the backbone assignment experiments was 0.15 mM; that of diubiquitin for the HD exchange experiments was 0.05 mM. Protein concentrations were determined by absorbance at 280 nm using a NanoDrop 2000c spectrophotometer (Thermo Fisher Scientific, Waltham, MA, USA). In the HD exchange experiments on native M1-linked diubiquitin, ^1^H-^15^N HSQC spectra were sequentially acquired at 310 K. Data processing was performed in NMRPipe [15] and the data were analyzed in CcpNmr Analysis [16].

### 2.4. Real-Time NMR Analysis

In the HD exchange experiments on M1-linked diubiquitin in the native state, the signal intensities *I*(*t*) of each ^1^H-^15^N cross-peak at time *t* after start of the HD exchange were fitted to the equation *I*(*t*) = *I*_0_ exp(−*k*_ex_ *t*), in which *I*_0_ is the initial signal intensity and *k*_ex_ is the HD exchange rate. Data fitting was performed by using the program GLOVE [17]. Errors were calculated by the Monte Carlo method. The obtained HD exchange rates are given in Table S1 in the Supplementary Materials.

## 3. Results

### 3.1. Backbone Assignment of Unfolded Ubiquitin in DMSO

**Figure 1:**
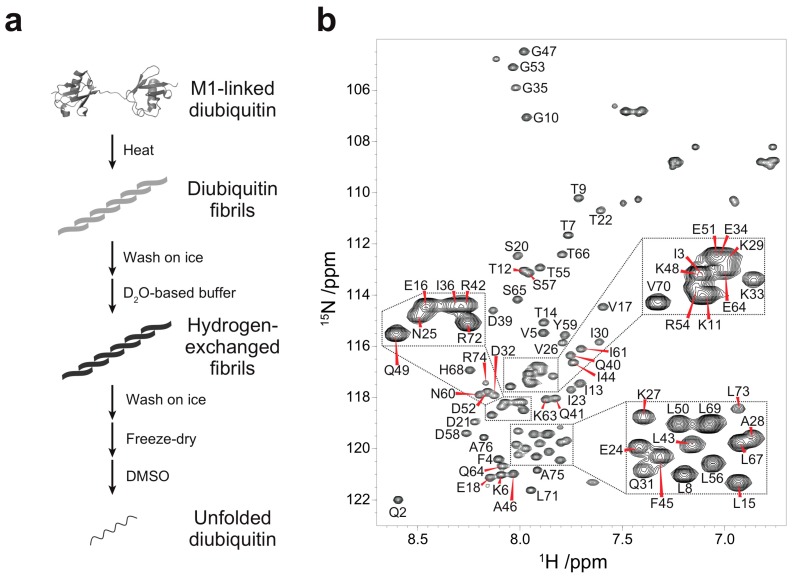
Quenched hydrogen-deuterium (HD) exchange experiments on M1-linked diubiquitin fibrils: (**a**) Strategy for the HD exchange experiments on M1-linked diubiquitin fibrils in dimethyl sulfoxide (DMSO). The M1-linked diubiquitin fibrils were formed by heat treatment, subsequently separated from residual native diubiquitin by centrifugation, and washed with ice-cold D_2_O-based buffer. The purified fibrils were suspended in the D_2_O-based buffer and incubated for 24 h. After the HD exchange, the fibrils were purified, freeze-dried, and dissolved in d_6_-DMSO containing 0.026% (*v/v*) d_1_-trifluoroacetic acid (TFA); (**b**) Assigned ^1^H-^15^N spectrum of the monoubiquitin G75A/G76A mutant in d_6_-DMSO containing 0.026% (*v/v*) d_1_-TFA.

Quenched hydrogen-deuterium (HD) exchange is often utilized to identify solvent-protected backbone amide protons of amyloid fibrils [18]. This method requires a sample to be dissolved and unfolded in dimethyl sulfoxide (DMSO), a solvent which has no exchangeable protons. To analyze solvent-protection of M1-linked diubiquitin fibrils by the quenched HD exchange method, we first recorded a ^1^H-^15^N HSQC spectrum of the monoubiquitin G75A/G76A mutant in DMSO containing 0.026% (*v/v*) trifluoroacetic acid (TFA) (Figure 1b) and assigned its HN, N, C_α_, and C_β_ chemical shifts (Figure S1 in the Supplementary Materials). A small amount of TFA was necessary to completely dissolve monoubiquitin in DMSO and we observed no changes in cross-peak intensities caused by TFA at this low concentration. All backbone cross-peaks were located in the narrow region between 7.5 and 8.7 ppm in the ^1^H dimension, characteristic for unstructured peptides and proteins (Figure 1b). This indicated that monoubiquitin had adopted a disordered structure in DMSO. Because the tertiary structure of each ubiquitin subunit in polyubiquitin is almost identical to that of monoubiquitin [1], the ubiquitin subunits in polyubiquitin are expected to be unfolded in DMSO similar to monoubiquitin. Therefore, we prepared fibrils of M1-linked diubiquitin whose distal or proximal subunit was selectively ^15^N-labeled and acquired the ^1^H-^15^N HSQC spectra of the fibrils unfolded in DMSO. Although the cross-peaks of several *N*-terminal and *C*-terminal residues were largely shifted with respect to the corresponding signals of monoubiquitin, no significant chemical shift differences were observed for the large majority of the ^1^H-^15^N cross-peaks (Figure S2 in the Supplementary Materials). These observations indicated that both subunits of M1-linked diubiquitin were unfolded in DMSO and that the cross-peak assignments of monoubiquitin in DMSO can be transferred to unfolded M1-linked diubiquitin.

### 3.2. Solvent-Protected Core of M1-Linked Diubiquitin Fibrils

**Figure 2:**
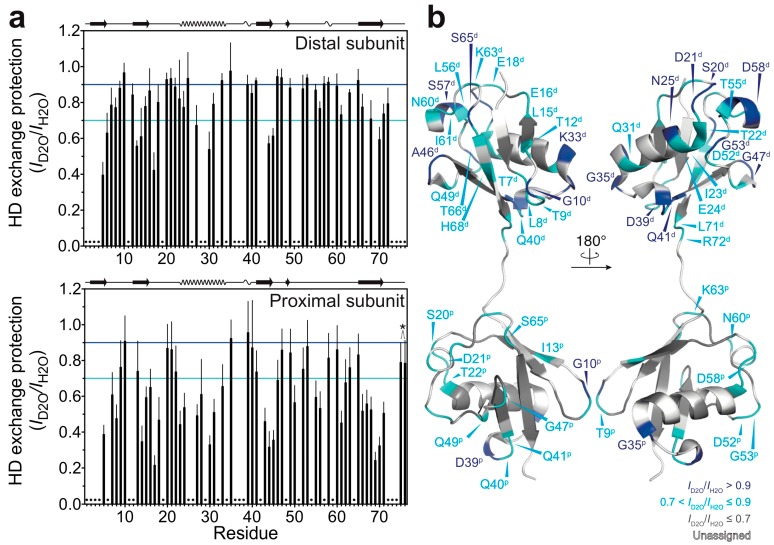
HD exchange profiles of M1-linked diubiquitin fibrils: (**a**) HD exchange protection was estimated from the ratio of peak intensities of the fibrils incubated in the D_2_O-based buffer (*I*_D2O_) to those of the fibrils incubated in the H_2_O-based buffer (*I*_H2O_): top, the distal subunit; bottom, the proximal subunit. The cyan and blue lines indicate HD exchange protection (*I*_D2O_/*I*_H2O_) values of 0.7 and 0.9, respectively. On the top of each graph, the secondary structure elements of natively folded M1-linked diubiquitin are schematically displayed (β-strand, α-helix, and 3_10_-helix) based on its crystal structure [Protein Data Bank (PDB) database entry 2W9N]. Error bars represent the standard deviation of three independent experiments. Residues indicated by dots were excluded from the analysis due to heavy peak overlap in the NMR spectra. The asterisk indicates the point mutations in ubiquitin (G75A/G76A); these two HD exchange protection values were excluded from the discussion; (**b**) Residues showing 0.7 < *I*_D2O_/*I*_H2O_ ≤ 0.9 (cyan) and *I*_D2O_/*I*_H2O_ > 0.9 (blue) are mapped on the crystal structure of M1-linked diubiquitin (PDB database entry 2W9N). Residues colored in grey show *I*_D2O_/*I*_H2O_ ≤ 0.7 and residues colored in white are excluded from the analysis due to peak overlap. The superscripts p and d indicate, respectively, the proximal and distal subunit of M1-linked diubiquitin.

By using the quenched HD exchange method, we investigated to what extent the amide protons of M1-linked diubiquitin fibrils are solvent-protected. The degree of HD exchange protection was estimated by comparison of the ^1^H-^15^N cross-peak intensities of M1-linked diubiquitin fibrils incubated in the D_2_O-based buffer with those incubated in the H_2_O-based buffer (see the Method section: Hydrogen-Deuterium Exchange Experiments). Interestingly, the distal subunits of M1-linked diubiquitin in the fibrils showed a higher overall solvent-protection as compared with the proximal subunit (Figure 2a). The mean ± standard deviation of the HD exchange protection values was 0.79 ± 0.15 for the distal subunit and 0.62 ± 0.20 for the proximal subunit. These results were surprising because the two ubiquitin moieties in M1-linked diubiquitin were expected to have similar solvent accessibilities due to their indistinguishable tertiary structures [19]. This asymmetry in HD exchange protection suggests that the distal subunit is more involved in the formation of a core structure in polyubiquitin fibrils as compared with the proximal subunit.

Furthermore, we found that the relatively solvent-protected residues are located in loop regions and at edges of secondary elements of the native structure of M1-linked diubiquitin (Figure 2b: residues colored in blue and cyan). In particular, the amide protons of G10, G35, and D39 (located in the loop between the β-strands β1 and β2, the edge of the α-helix, and the first 3_10_-helix in the native structure, respectively) were highly solvent-protected in both ubiquitin subunits in the structure of the fibrils. In the distal subunit, S20 and D21 (located in the loop between the β-strand β2 and the α-helix), N25 and K33 (the edges of the α-helix), Q41 (first residue of the β-strand β3), A46 and G47 (loop between β3 and β4), G53 (turn between the β-strand β4 and the second 3_10_-helix), S57 and D58 (the second 3_10_-helix) were also highly solvent-protected in the diubiquitin fibrils (note that parentheses indicate the secondary structure in *natively folded* diubiquitin). 

**Figure 3:**
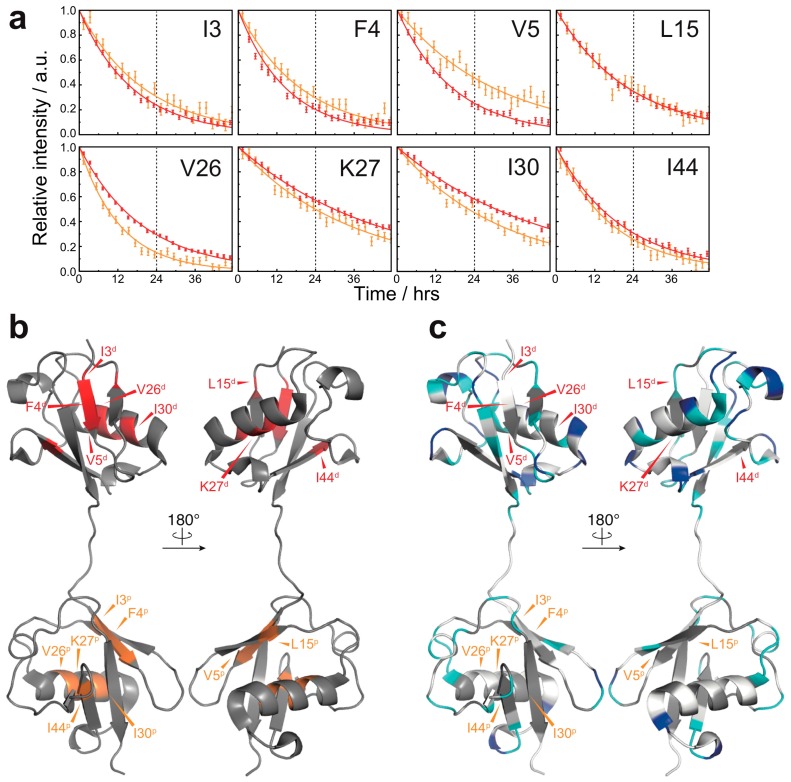
HD exchange profiles in the native state of M1-linked diubiquitin: (**a**) HD exchange profiles of the residues showing the lowest HD exchange rates in the native state (red, the proximal subunit; orange, the distal subunit). For all other residues, the amide proton was completely exchanged to deuterium by the time of 24 h. Errors were estimated from the spectral noise; (**b**) Mapping of the residues showing the lowest HD exchange rates on the crystal structure of M1-linked diubiquitin (PDB database entry 2W9N). The superscripts p and d indicate, respectively, the proximal and distal subunit of M1-linked diubiquitin; (**c**) Comparison of the HD exchange protected regions between the native state and fibrils. The most protected residues in the native state do not correspond to highly protected residues in the fibrils. The color code is the same as defined in Figure 2b.

By contrast, most of the amide protons in natively folded diubiquitin were completely exchanged to deuterium within 24 h. This suggests that the overall solvent-accessibility was drastically reduced owing to fibril formation. Hydrogen exchange of several residues (I3, F4, V5, L15, V26, K27, I30, and I44) of natively folded diubiquitin, however, continued past 24 h (Figure 3a,b). Although a few residues (I3, F4, and V26 of both subunits) had to be excluded from the analysis due to peak overlap, for the majority of the residues (K27 and I30 of the distal subunit; V5, K27, and I44 of the proximal subunit), the estimated HD exchange protection values were comparable between the native state and the fibrils (Figure S3 in the Supplementary Materials). This result suggests that the structure of native ubiquitin can partially remain intact in the diubiquitin fibrils. Compared to diubiquitin fibrils, however, several residues (V5, L15, and I44 of the distal subunit; L15 of the proximal subunit) were less solvent-protected in the native state (Figure S3 in the Supplementary Materials); in addition, I30 of the proximal subunit was more solvent-protected in the native state (Figure S3 in the Supplementary Materials). Thus, the change in solvent-accessibility of these particular residues may arise from a more complicated structural rearrangement in the course of fibril formation. Taken together, in the formation of fibrils, inter-molecular interactions via many residues are formed presumably due to a combination of hydrogen bonds, electrostatic, and hydrophobic interactions. In contrast, the diubiquitin fibrils may also retain a part of the native structure of ubiquitin, which might enable binding by recognizing proteins.

**Figure 4:**
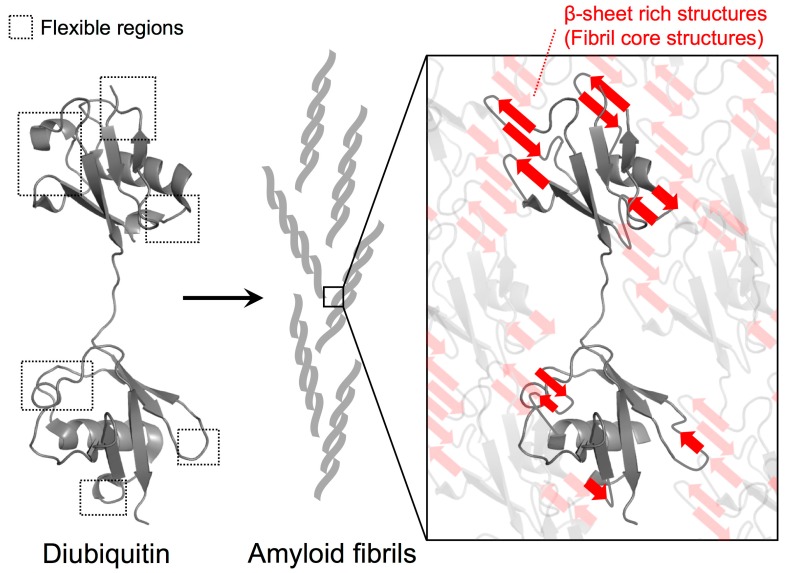
Proposed model for polyubiquitin fibril core structure formation. Based on the HD exchange profiles in M1-linked diubiquitin fibrils, the intrinsically flexible regions of the ubiquitin subunits were highly solvent-protected in the fibrils, suggesting that they contribute to the formation of β-sheet rich structures of the fibrils. According to the protection values, the contribution of the distal subunits of diubiquitin to formation of fibril core structures may be more pronounced than the proximal subunit. By contrast, the intrinsic hydrophobic structures such as the I44-centered hydrophobic surface can remain intact in the fibrils.

In addition to the formation of new interactions among diubiquitin molecules in the fibrils, the identified solvent-protected residues of the fibrils could also be involved in the formation of β-sheet rich structures. In a β-sheet, the amide protons are expected to be rather solvent-protected because they engage in hydrogen bonding between β-strands. Because we previously observed the formation of β-sheet rich structures in M1-linked diubiquitin fibrils by circular dichroism [4], relatively flexible structures such as loops and edges of secondary elements might be converted into intra- or inter-molecular β-sheet structures in which these amide protons are more solvent-protected (Figure 4). Recently, we observed that the chemical shifts of the side chains of residues located in flexible regions (K11, P19, K48, Q62, and K63), the edges of the α-helix (E24 and K33), and the first 3_10_-helix (P37, P38, and D39) undergo changes during the formation of polyubiquitin fibrils from the native structure [20]. This suggests that inter-molecular associations and secondary structure changes in these regions take place in the course of fibril formation.

## 4. Discussion

HD exchange experiments allow the identification of solvent-protected backbone amide protons. Here, the observed HD exchange protection profiles suggest that polyubiquitin fibrils are formed by the association between intrinsically flexible regions rather than by drastic rearrangements of secondary structure elements (Figure 2b). Because we previously observed that polyubiquitin fibrils can be recognized by ubiquitin-adaptor proteins such as p62 and NBR1, the hydrophobic surface centered on I44 for ubiquitin recognition may remain intact in the fibrils and thus stay exposed to solvent similar to native ubiquitin [21,22]. This is consistent with the observation that the HD exchange protection of polyubiquitin fibrils was not particularly pronounced in the I44-centered hydrophobic surface (Figure 2 and Figure 3c); rather, these exchange rates were comparable to the native state (Figure S3 in the Supplementary Materials). In addition, unlike intrinsically disordered amyloid-prone proteins such as amyloid β and α-synuclein, natively folded amyloid-forming proteins including β2-microglobulin and superoxide dismutase 1 (SOD1) have more complex fibril formation pathways [23]. In the amyloid formation of folded proteins, partial structural rearrangements and/or conversion of intrinsically flexible structures to β-sheet structures can initiate oligomerization and fibril formation. In particular, the flexible loops of SOD1 play an essential role in the structures of the oligomeric states and fibrils [24]. This analogy to SOD1 may illustrate why in polyubiquitin fibrils, intrinsically flexible regions were found to be highly solvent-protected (Figure 2b and Figure 4).

Taken together, the HD exchange experiments provided insight into the molecular interactions and structural changes in fibril formation of polyubiquitin. However, the detailed structure of polyubiquitin fibrils remains to be elucidated; therefore, future studies will aim to determine the atomic-level structure of polyubiquitin fibrils by solid-state NMR spectroscopy or electron microscopy, and—in combination with the HD exchange profiles in this study—derive the formation mechanism of polyubiquitin fibrils.

## Supplementary Materials

The following are available online at http://www.mdpi.com/2073-4360/10/3/240/s1, Figure S1: Chemical shift assignments of unfolded ubiquitin in dimethyl sulfoxide (DMSO), Figure S2: Subtle chemical shift differences between unfolded monoubiquitin and each subunit of M1-linked diubiquitin fibrils dissolved in DMSO, Figure S3: Comparison of hydrogen-deuterium (HD) exchange protection values between native M1-linked diubiquitin and its fibrils, Table S1: HD exchange rates of diubiquitin in the native state.
