# Supplementary material for: Hydrogen-Deuterium Exchange Profiles of Polyubiquitin Fibrils
Source: Polymers (Basel). 2018 Feb 27;10(3):240. doi: 10.3390/polym10030240 (PMC6414897; doi:10.3390/polym10030240)
Supplement: Supplementary file 1 [file polymers-10-00240-s001.pdf]

Supplementary Materials

Hydrogen-deuterium exchange profiles of polyubiquitin fibrils

Daichi Morimoto, Ryo Nishizawa, Erik Walinda, Shingo Takashima, Kenji Sugase*,

and Masahiro Shirakawa*

Supplementary Figures

Residue	HN /ppm	N /ppm	C_α /ppm	C_β /ppm
1	–	–	54.69	34.15
2	8.59	122.00	55.33	30.91
3	7.95	117.10	59.73	39.92
4	8.10	120.40	56.50	40.30
5	7.89	115.48	60.54	33.82
6	8.09	121.03	55.39	34.18
7	7.76	111.67	60.89	69.77
8	7.92	120.31	54.27	43.53
9	7.71	110.22	61.37	69.88
10	7.97	107.06	45.09	–
11	7.94	117.44	55.12	34.67
12	7.98	113.06	61.31	69.64
13	7.71	117.45	59.88	40.02
14	7.88	115.06	61.24	69.76
15	7.80	120.45	53.94	44.05
16	8.07	118.22	54.97	30.17
17	7.59	114.46	60.11	33.89
18	8.15	121.14	52.92	29.39
19	–	–	62.35	32.16
20	8.01	112.47	58.28	64.79
21	8.22	118.95	52.67	38.80
22	7.61	110.70	61.28	69.79
23	7.75	117.71	60.43	39.45
24	8.02	119.84	55.31	30.07
25	8.09	118.36	52.96	39.60
26	7.79	115.87	61.82	32.97
27	8.01	119.31	56.16	33.46
28	7.78	119.69	51.70	20.76
29	7.89	116.81	55.89	33.89
30	7.61	115.83	60.43	39.52
31	8.01	120.23	55.42	30.92
32	8.13	117.92	52.74	38.95
33	7.84	117.18	55.48	34.21
34	7.91	116.78	55.16	30.46
35	8.02	105.90	44.56	–
36	8.01	118.19	57.44	39.28
37	–	–	–	–
38	–	–	63.00	31.95
39	8.13	114.61	53.11	38.48
40	7.75	116.35	55.41	30.93

Residue	HN /ppm	N /ppm	C_α /ppm	C_β /ppm
41	7.83	118.04	55.55	30.72
42	7.99	118.18	55.29	32.09
43	7.90	119.79	54.24	43.54
44	7.74	116.63	60.06	39.65
45	7.97	120.00	56.58	40.14
46	8.03	120.98	51.37	21.29
47	7.98	104.48	44.95	–
48	7.96	117.08	55.12	34.64
49	8.14	118.67	55.31	30.59
50	7.93	119.43	54.11	43.70
51	7.92	116.78	54.87	30.37
52	8.16	117.78	52.63	39.15
53	8.04	105.09	45.09	–
54	7.96	117.32	54.96	32.48
55	7.90	112.92	61.26	69.77
56	7.86	120.12	54.06	43.84
57	7.96	113.15	57.78	64.98
58	8.26	119.41	52.88	38.92
59	7.78	115.56	57.47	39.69
60	8.20	117.89	52.74	39.92
61	7.70	116.09	60.27	39.70
62	8.09	120.68	55.83	30.40
63	7.87	118.07	55.46	34.23
64	7.90	116.98	54.88	30.57
65	8.01	114.18	57.90	64.77
66	7.79	112.41	61.40	69.35
67	7.80	119.76	54.40	43.39
68	8.25	116.93	54.55	29.77
69	7.87	119.43	54.24	43.97
70	8.05	117.56	61.00	33.34
71	7.95	121.62	53.95	43.83
72	7.98	118.49	55.05	31.96
73	7.80	119.17	53.62	44.22
74	8.17	117.44	54.82	31.06
75	7.92	120.83	50.95	21.25
76	8.18	119.56	50.63	20.21

Figure S1. Chemical shift assignments of unfolded ubiquitin in dimethyl sulfoxide (DMSO). ^{1}H chemical shifts were referenced with respect to sodium 2,2-dimethyl-2-silapentane-5-sulfonate (DSS) and both ^{13}C and ^{15}N chemical shifts were calibrated indirectly.

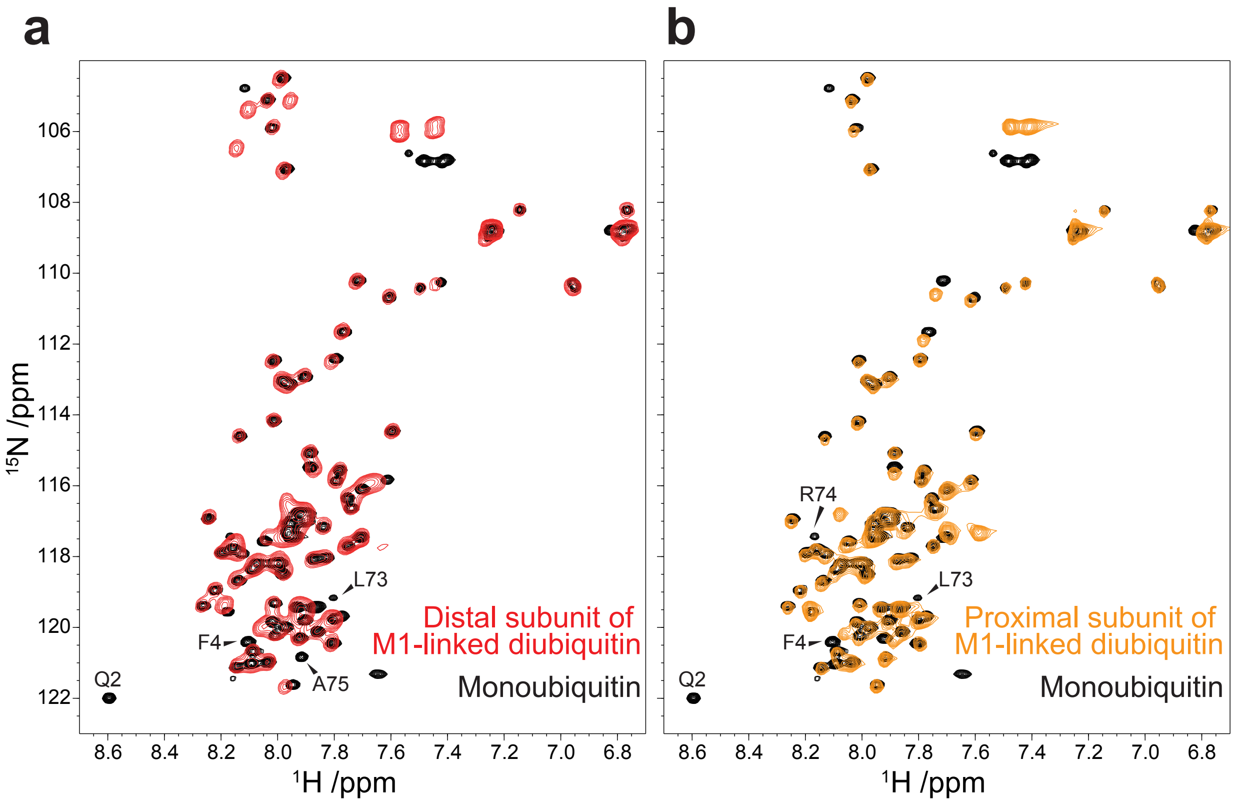

Figure S2. Subtle chemical shift differences between unfolded monoubiquitin and each subunit of M1-linked diubiquitin fibrils dissolved in DMSO. ^{1}H-^{15}N HSQC spectra of monoubiquitin G75A/G76A mutant (black), distal (**a**, red), and proximal (**b**, orange) subunits of M1-linked diubiquitin fibrils dissolved in d_6-DMSO containing 0.026% (v/v) d_1-TFA at 298 K. The monoubiquitin and diubiquitin fibrils appeared to be completely unfolded in the DMSO solution. On average, the ^{15}N NMR linewidth (full-width at half-height) of the diubiquitin cross-peaks was 2.4-fold larger than that of the monoubiquitin cross-peaks. This increase is caused mainly due to the increase in the molecular weight from monoubiquitin to diubiquitin. The cross-peaks of the N-terminal (Q2 and F4) and C-terminal (L73, R74, and A75) residues were shifted presumably due to the presence of an additional N-terminal sequence in the distal subunit, diubiquitin formation, or conformational exchanges in the DMSO solution; however, no large differences were observed for the remaining signals.

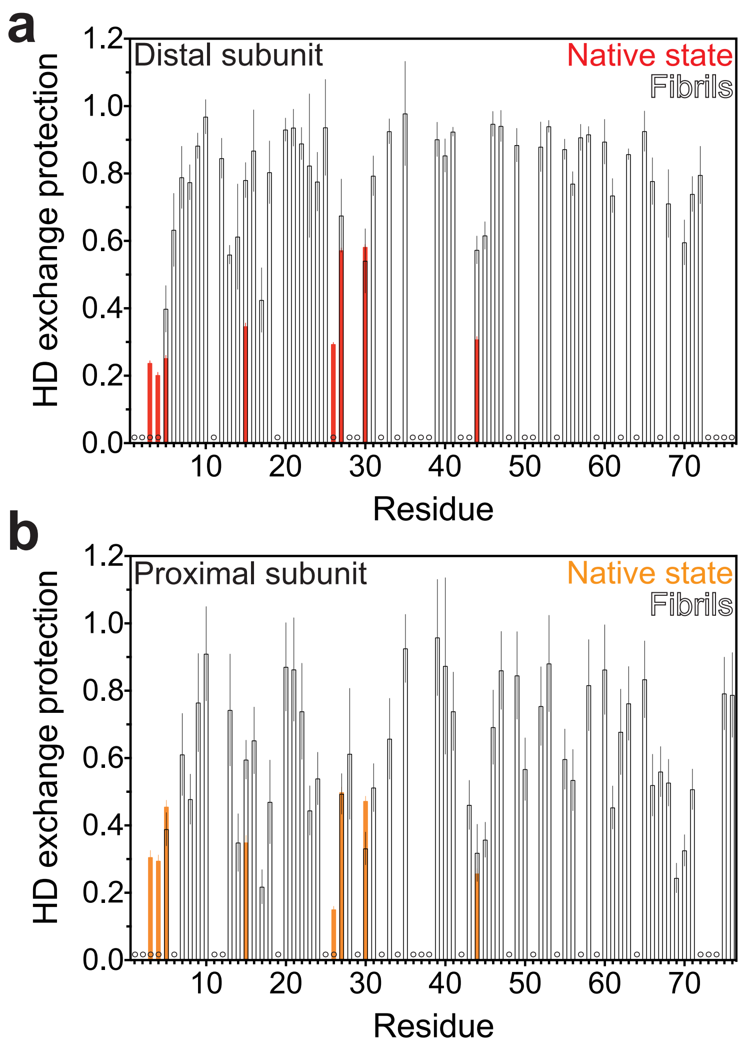

Figure S3. Comparison of hydrogen-deuterium (HD) exchange protection values between native M1-linked diubiquitin and its fibrils. HD exchange protection of the distal (**a**, red bars) and proximal subunits (**b**, orange bars) of diubiquitin in the native state was estimated from the normalized signal intensity of each cross-peak in the ^{1}H-^{15}N spectra at 24 hours after start of the HD exchange: the signal intensity at the start ($t = 0$) is set to 1. Each signal intensity at 24 hours after the start was calculated from the obtained exchange rates (Table S1 in the Supplementary Materials). With the exception of I3, F4, V5, L15, V26, K27, I30, and I44, all the amide protons in native diubiquitin were completely exchanged to deuterium within 24 hours. For comparison, the HD exchange protection profiles of diubiquitin fibrils (Figure 2a) are also displayed (white bars). The circles in each graph indicate residues that had to be excluded from the analysis of the fibril HD exchange profile due to heavy peak overlap in the spectra. Errors in the HD exchange protection values of diubiquitin in the native state were obtained by error propagation.

Supplementary Table

Table S1. HD exchange rates of diubiquitin in the native state.

Residue	k_{ex} / 10^{-2} $hour^{-1}$ (distal subunit)	k_{ex} / 10^{-2} $hour^{-1}$ (proximal subunit)
I3	6.0 ± 0.1	4.9 ± 0.2
F4	6.7 ± 0.2	5.1 ± 0.2
V5	5.7 ± 0.1	3.3 ± 0.1
L15	4.4 ± 0.2	4.4 ± 0.2
V26	5.1 ± 0.1	7.9 ± 0.2
K27	2.3 ± 0.1	2.9 ± 0.1
I30	2.3 ± 0.1	3.1 ± 0.1
I44	4.9 ± 0.1	5.7 ± 0.2

For each cross-peak in the ^{1}H-^{15}N spectra, the signal intensities $I(t)$ at time t after start of the HD exchange were fitted to the equation $I(t) = I_0 \exp(-k_{ex}\, t)$, in which I_0 is the initial signal intensity and k_{ex} is the HD exchange rate. Errors indicate the standard deviation as obtained by Monte-Carlo simulation (100 iterations).
